# Supplementary material for: A novel application tunnel in combination with medical training reduces stress induced by frequent intraperitoneal injections and blood draws in mice
Source: PLoS One. 2026 May 7;21(5):e0341404. doi: 10.1371/journal.pone.0341404 (PMC13152133; doi:10.1371/journal.pone.0341404)
Supplement: S1 Table — (PDF) [file pone.0341404.s001.pdf]

Table S1. Overview of experimental groups

#	Disease model	Treatment	Training regimen	Injection method	Cage	Mouse	LtI	Corticosterone	Tracking	Pathology
**1**	Untreated (n=6)	None	None	None	C1	M1	x	x		
					C2	M2	x	x		
						M3	x	x		
						M4	x	x		
					C3	M5	x	x		
						M6	x	x		
					C4	M7	x		x	
						M8	x		x	
						M9	x		x	
						M10	x		x	
						M11	x		x	
					C5	M12	x		x	
						M13	x		x	
						M14	x		x	
					C6	M15	x		x	
						M16	x		x	
						M17	x		x	
						M18	x		x	
						M19	x		x	
**2**	CCl_4 (n=15)	Ip-injections twice a week starting at week 8 (CCl_4) and daily at week 18 (Immunotherapy)	None	Conventional	C7	M20		x		
						M21		x		
					C8	M22		x		
						M23				
						M24		x		
					C9	M25		x	x	
						M26		x	x	
						M27		x	x	
						M28		x	x	
						M29		x	x	
					C10	M30			x	
						M31			x	
						M32			x	
						M33			x	
						M34			x	
**3**	CCl_4+T (n=10)	Ip-injections twice a week starting at week 8 (CCl_4) and daily at week 18 (Immunotherapy)	None	Tunnel	C11	M35		x		x
						M36		x		
						M37		x		
						M38				x
					C12	M39		x		
						M40		x		
						M41				
					C13	M42				x
						M43				x
						M44				x
**4**	CCl_4+MT (n = 23)	Ip-injections twice a week starting at week 8 (CCl_4) and daily at week 18 (Immunotherapy)	Basic training from week 6, followed by continuous training (2 sessions every other week)	Conventional	C14	M45	x			
						M46	x			
					C15	M47	x	x		
						M48	x	x		
						M49	x	x		
						M50	x	x		
						M51	x	x		
					C16	M52	x			
						M53	x			
						M54	x	x		
					C17	M55	x			
						M56	x			
						M57	x			
						M58	x			
						M59	x			
					C18	M60	x	x		
						M61	x	x		
						M62	x	x		
						M63	x	x		
					C19	M64	x			
						M65	x			
						M66	x			
						M67	x			

#	Disease model	Treatment	Training regimen	Injection method	Cage	Mouse	LtI	Corticosterone	Tracking	Pathology
5	CCl4+MT+T (n=14)	Ip-injections twice a week starting at week 8 (CCl4) and daily at week 18 (Immunotherapy)	Basic training from week 6, followed by continuous training (2 sessions every other week)	Tunnel	C20	M68	x	x		
						M69	x	x		
						M70	x	x		
					C21	M71	x	x	x	
						M72	x	x	x	
						M73	x	x	x	
						M74	x	x	x	
						M75	x	x	x	
					C22	M76	x			x
						M77	x			x
					C23	M78	x	x	x	
						M79	x	x	x	
						M80	x		x	
						M81	x		x	
6	WD (n=9)	WD from week 8, daily ip-injections at week 24 (Immunotherapy)	None	Conventional	C24	M82		x		
						M83		x		
						M84		x		
					C25	M85		x		
						M86		x		
						M87		x		
						M88		x		
						M89		x		
						M90		x		
7	WD+MT, early (n=29)	WD from week 8, daily ip-injections at week 24 (Immunotherapy)	Basic training from week 6, followed by continuous training (2 sessions every other week)	Conventional	C26	M91	x			
						M92	x			
						M93	x			
						M94	x			
						M95	x			
						M96	x			
					C27	M97	x			
						M98	x			
						M99	x			
						M100	x			
					C28	M101	x	x		
						M102	x	x		
						M103	x	x		
						M104	x	x		
					C29	M105	x	x		
						M106	x			
						M107	x			
						M108	x			
						M109	x	x		
					C30	M110	x			
						M111	x	x		
						M112	x			
						M113	x			
						M114	x	x		
					C31	M115	x	x		
						M116	x			
						M117	x			
						M118	x			
						M119	x	x		
8	WD+MT late (n= 21)	WD from week 8, daily ip-injections at week 24 (Immunotherapy)	Basic training from week 22 (2 weeks before ip-injections begin)	Conventional	C32	M120	x	x		
						M121	x	x		
						M122	x	x		
						M123	x	x		
						M124	x	x		
					C33	M125	x	x		
						M126	x	x		
						M127	x	x		
						M128	x	x		
					C34	M129	x			
						M130	x			
						M131	x			
					C35	M132	x			
						M133	x			
						M134	x			
					C36	M135	x			
						M136	x			
						M137	x			
						M138	x			
						M139	x	x		
						M140	x			
