# Supplementary material for: A novel application tunnel in combination with medical training reduces stress induced by frequent intraperitoneal injections and blood draws in mice
Source: PLoS One. 2026 May 7;21(5):e0341404. doi: 10.1371/journal.pone.0341404 (PMC13152133; doi:10.1371/journal.pone.0341404)
Supplement: S2 Table — (PDF) [file pone.0341404.s002.pdf]

Table S2. Overview of the basic training program. 5 sessions are spread over 2 weeks with 2-3 days between each session. In general, signs of stress or anxiety, like the latency to interact or defecation/urination, should decrease over the training sessions and stay low.

Training day	Training program	Important considerations for handler behavior	Criteria of success	response to animal behavior
**1 (Fig.2a)**	Handler opens the cage, reaches the gloved hand inside and waits for voluntary interaction. If possible, handler tries to carefully touch the mouse.	• Reach hand in one corner rather than the middle of the cage to not pressure the animals.<br>• Hold hand still and wait for voluntary interaction	Animals approach hand voluntarily	If no voluntary interaction occurs within 5 minutes, session should be terminated and repeated on the next day.
**2 (Fig.2b)**	Handler opens the cage and directs the mice one by one into handling tunnels, which will be lifted. The handler closes both ends of the tunnel with the hands to prevent animals from walking back out and holds handling tunnel in the air for 30 seconds, before placing the mice back into their home cages.	• Guiding the mice into the tunnel can take longer for some mice in the beginning, e.g. 30 -60 s.<br>• When guiding the mice into the tunnel, it's important to not "chase" the mice with the tunnel and to not trap them in a corner to force them to enter the tunnel. This would lead to negative associative sensitization.	Mice enter tunnel without force.	• If mice refuse to enter the tunnel, terminate session after 2min.<br>• In the beginning, mice often show signs of anxiety in the tunnel, such as defecation and urination. This is not a reason to terminate the training session but this type of behavior should decrease with training progression.
**3 (Fig.2c)**	Similar procedure as on day two, but the handling tunnels are used to transfer the mice onto a Vetbed outside of the cage. After being transferred, the handler tries to touch the animals gently for about 30 seconds, before transferring them back into the cage using the handling tunnel.	• Aim to let the animals get in contact with the handler first (e.g. sniffing) before touching.<br>• Because touching the back area can cause more sensitive reactions, it is suggested to start with the head area of the animals.	• Animals leave tunnel voluntarily and explore Vetbed<br>• Animals tolerate brief touching	• The mice should leave the tunnel voluntarily. If not, the handler can slightly increase the angle from the tunnel to the vet bed so that the mouse slides out very gently. In our experience, this works with less stress if the mouse is allowed to slide out caudal end first.<br>• If animals act nervous and try to escape, it can be helpful to offer them the tunnel, so they may hide for a moment before being let out onto the VetBed again
**4 (Fig.2d)**	Same as on day 3, with a focus on touching the mice in different areas (eg. neck, back, tail).	• Aim to let the animals approach the handler first before touching.	• Animals show less avoidant behavior (eg. flight or freezing) when being touched<br>• Animals show less signs of defecation or urination	
**5 (Fig.2e)**	Training of the handling for intraperitoneal injection: either conventional scruffing or directing the mice into the injection tunnel and holding the tail/sacrum for a few seconds. No actual injection is performed.	• Use a syringe without needle to touch the mice in the lower caudal area where injection would normally be performed<br>• Do not restrain the animals for too long (about the time that would be needed for performing an injection)	• Mice enter injection tunnel voluntarily/can be guided in easily	• Mice may show signs of anxiety such as defecation and urination (especially when being scruffed). This should decrease with training progression.<br>• If mice cannot be guided into the injection tunnel easily, terminate session after 5min and repeat on the following day.
