# Supplementary figures and images for: A novel application tunnel in combination with medical training reduces stress induced by frequent intraperitoneal injections and blood draws in mice
Source: PLoS One. 2026 May 7;21(5):e0341404. doi: 10.1371/journal.pone.0341404 (PMC13152133; doi:10.1371/journal.pone.0341404)

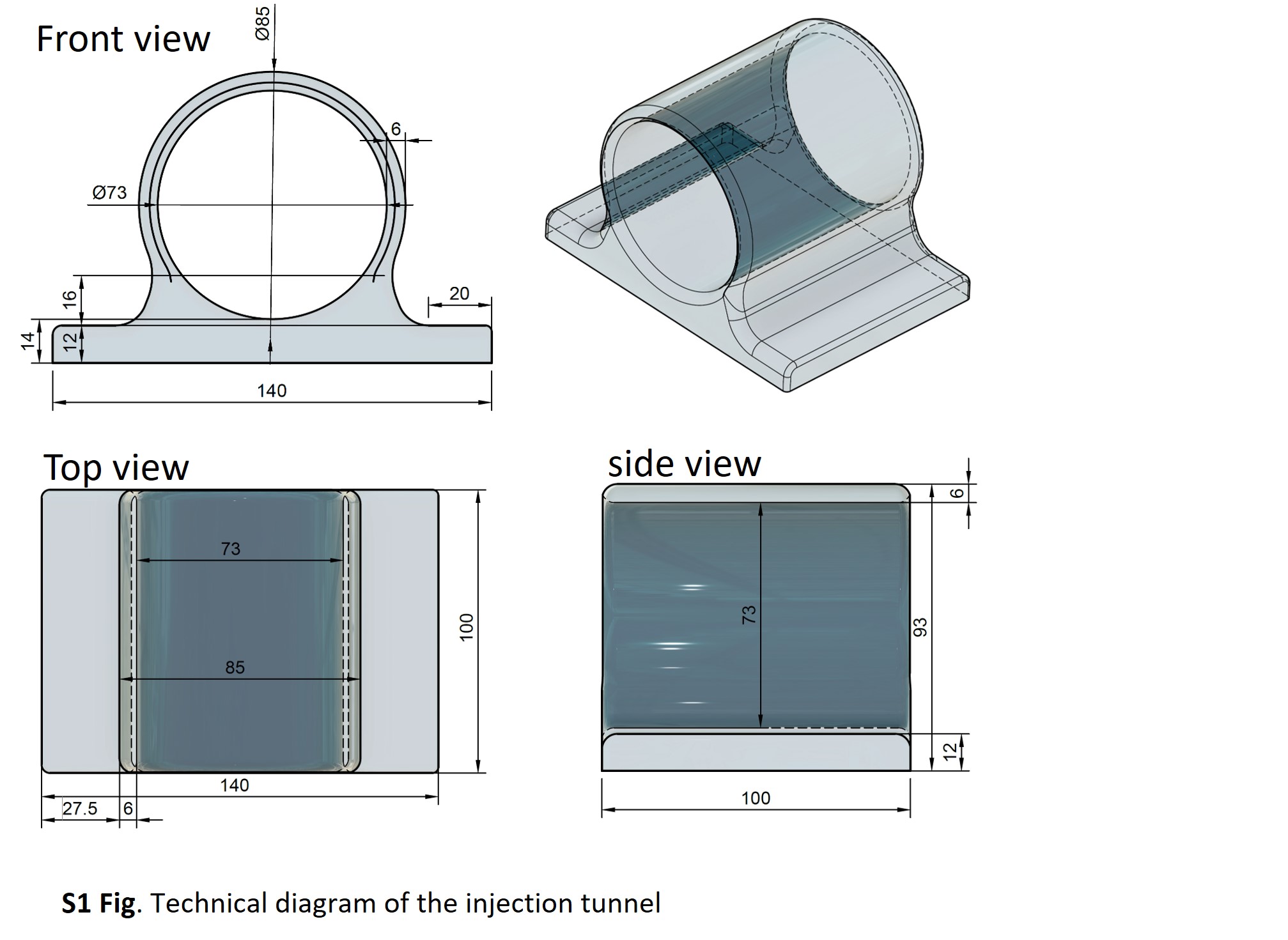

Supplement: S1 Fig — (JPG) [file pone.0341404.s003.jpg]

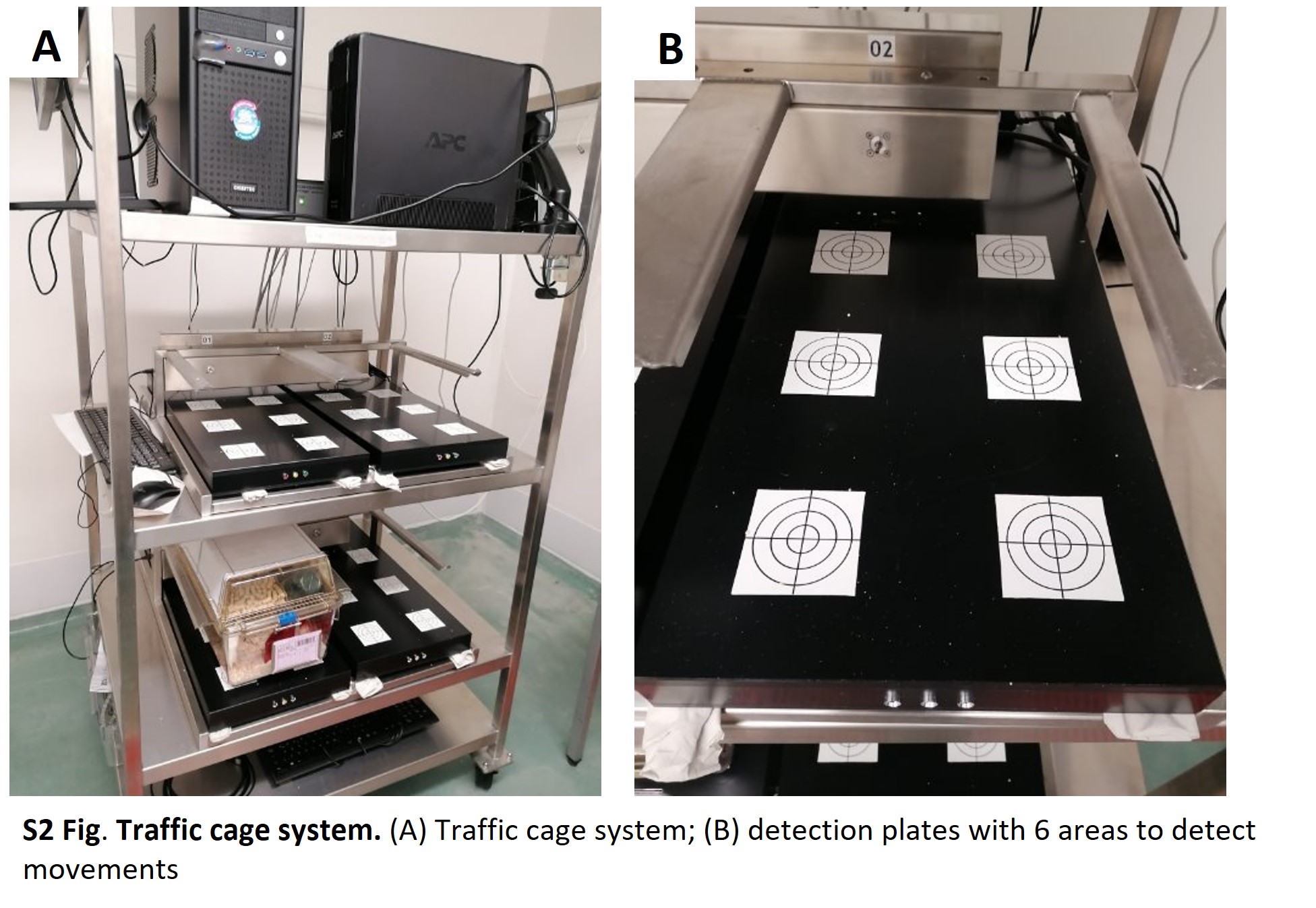

Supplement: S2 Fig — (JPG) [file pone.0341404.s004.jpg]

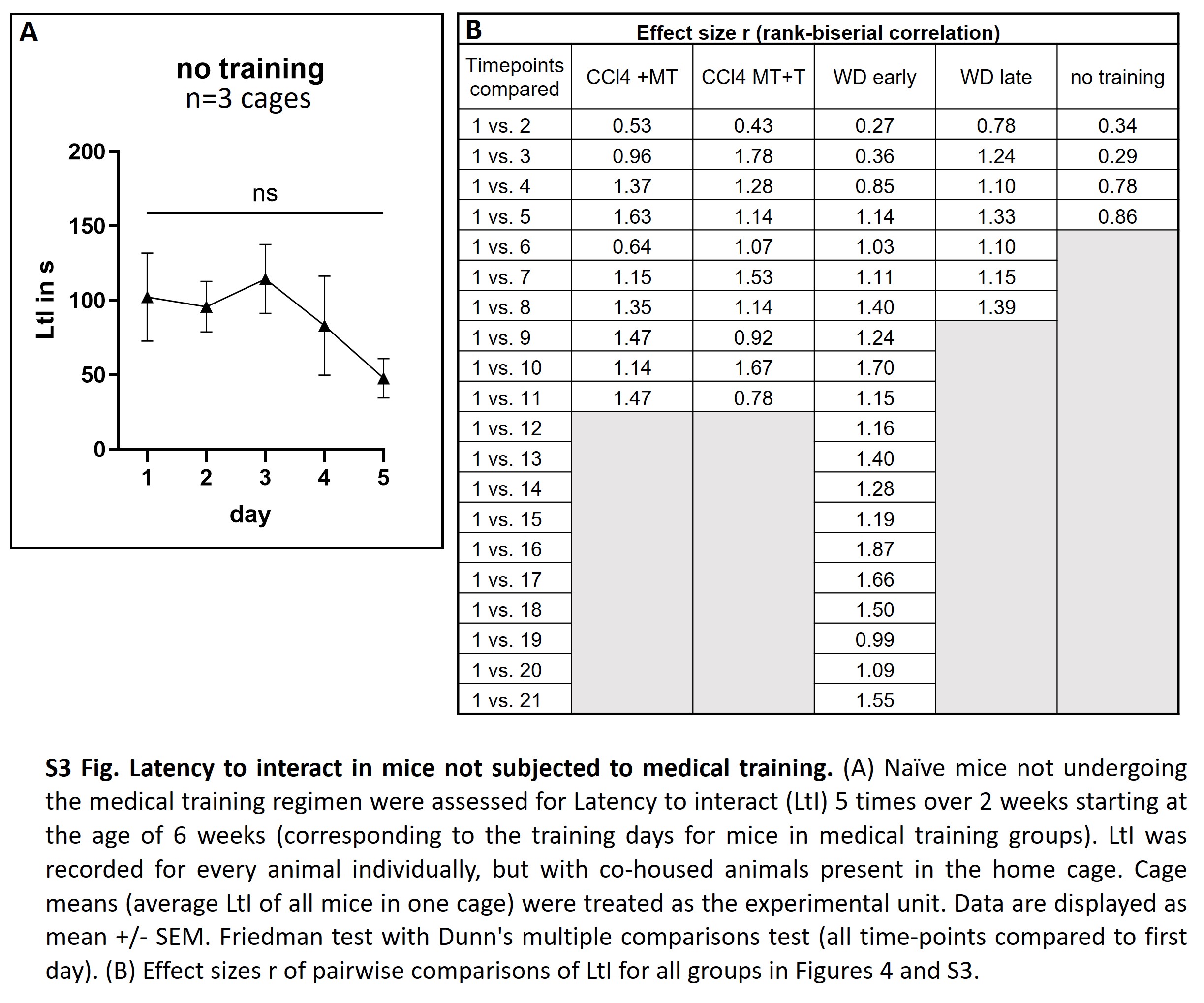

Supplement: S3 Fig — (JPG) [file pone.0341404.s005.jpg]

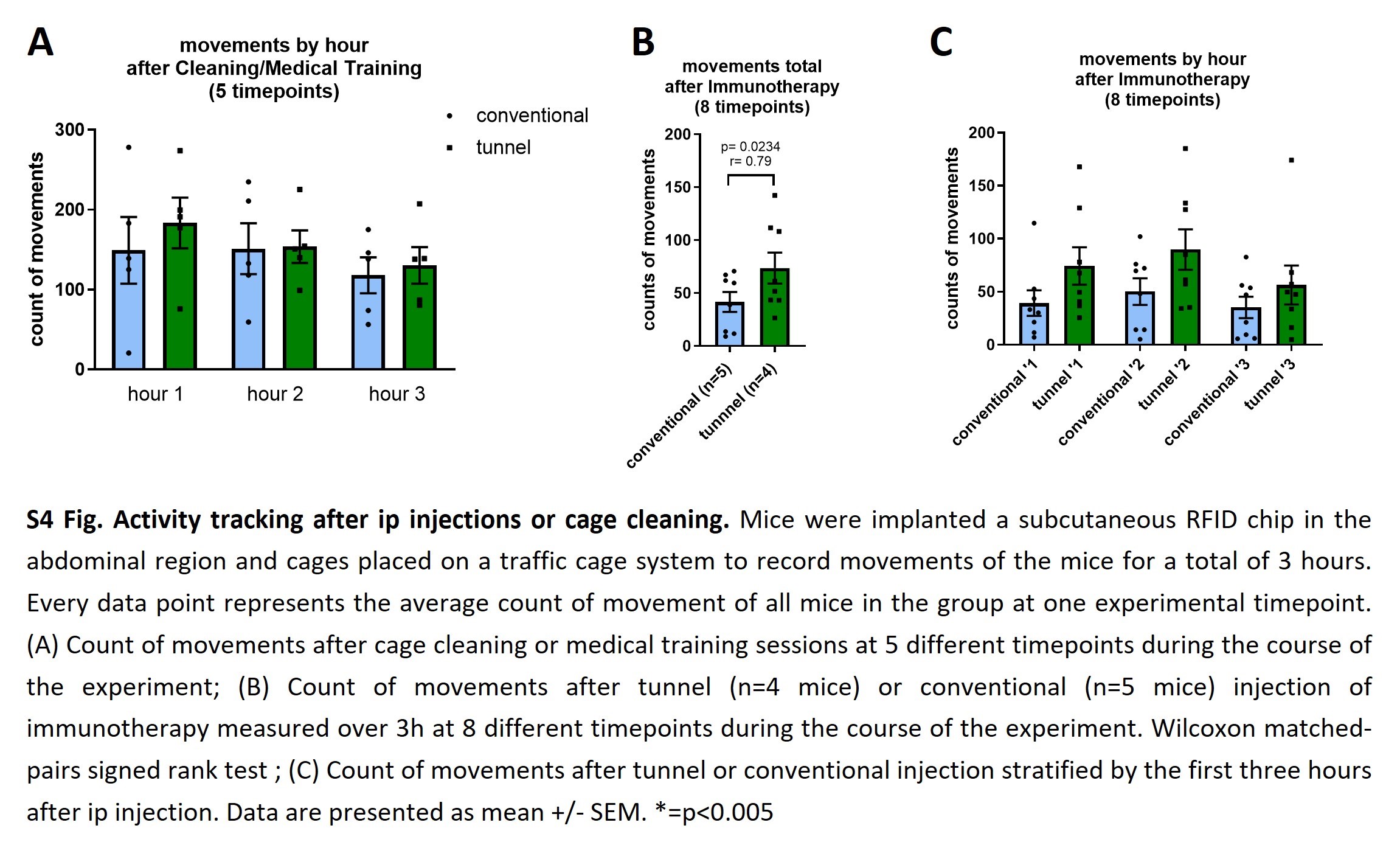

Supplement: S4 Fig — (JPG) [file pone.0341404.s006.jpg]

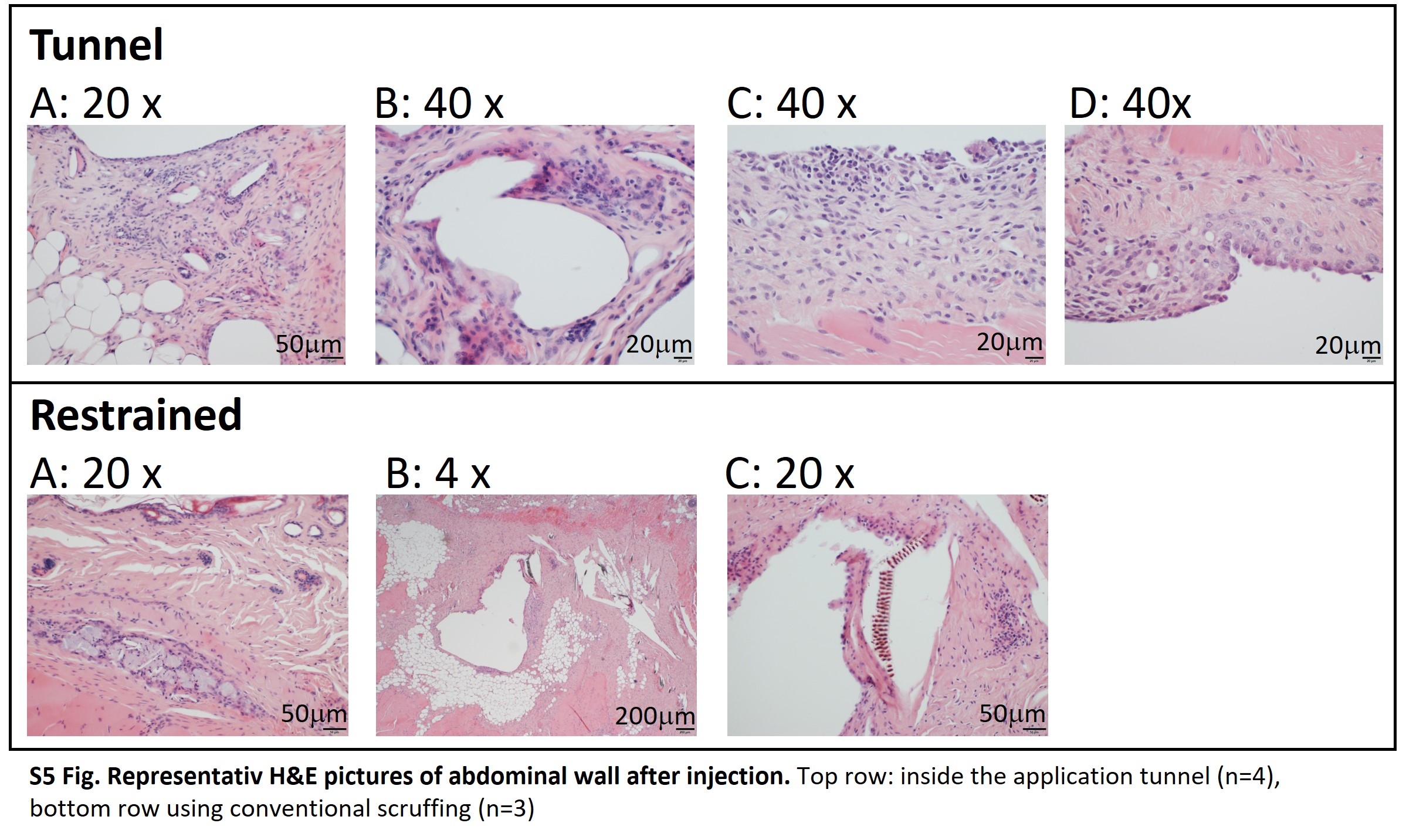

Supplement: S5 Fig — (JPG) [file pone.0341404.s007.jpg]

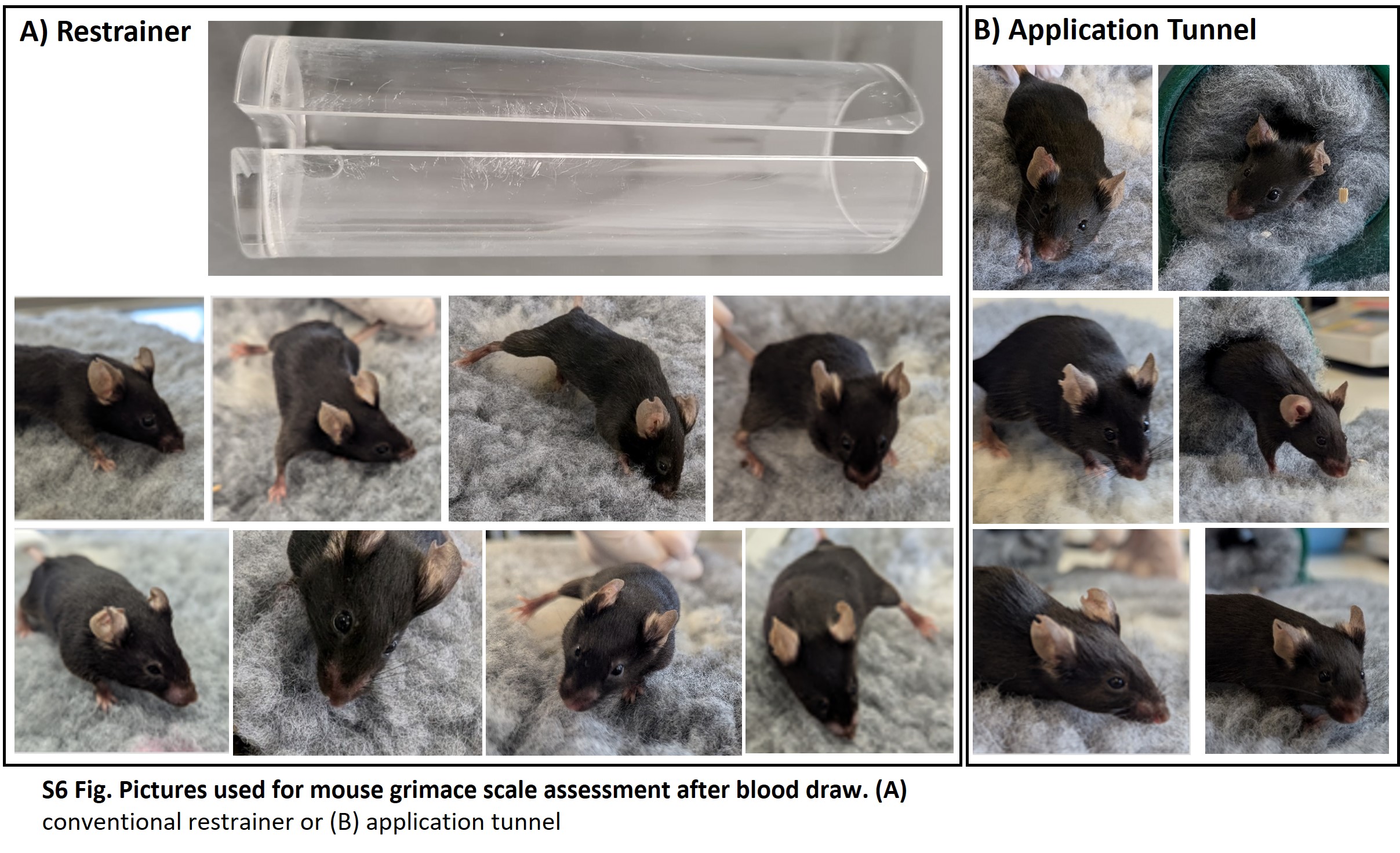

Supplement: S6 Fig — (JPG) [file pone.0341404.s008.jpg]

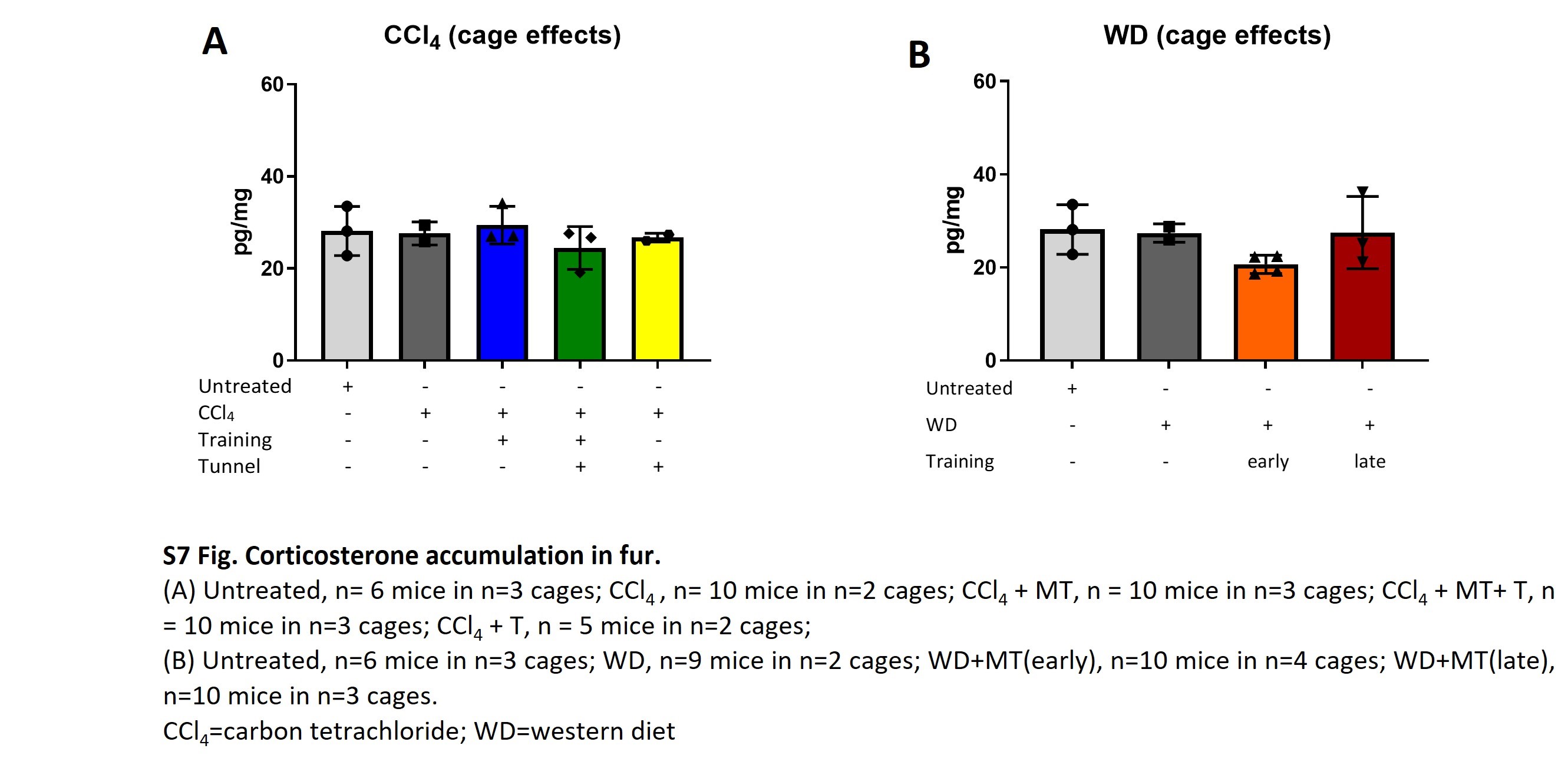

Supplement: S7 Fig — (JPG) [file pone.0341404.s009.jpg]
