# Supplementary material for: A novel application tunnel in combination with medical training reduces stress induced by frequent intraperitoneal injections and blood draws in mice
Source: PLoS One. 2026 May 7;21(5):e0341404. doi: 10.1371/journal.pone.0341404 (PMC13152133; doi:10.1371/journal.pone.0341404)
Supplement: Attachment — Submitted filename: Schlutt et al_response to reviewers.pdf [file pone.0341404.s012.pdf]

Thank you for evaluating our manuscript PONE-D-26-00796.
Please find our answers to the reviewers´ comments below:

Reviewer #1: The authors report on a novel tunnel designed for routine applications (injections and blood draws) that can reduce handling stress.
I have some concerns regarding statistical analyses.

1. The authors correctly use cage as the experimental unit by averaging measurements within cages (avoiding pseudoreplication), however, the sample sizes appear quite small (N=3-8 cages per group) with unequal group sizes.
The authors state they used the Shapiro-Wilk test to determine whether to use parametric or non-parametric tests. Shapiro-Wilk test has low power to check for normality when sample size is small. With small sample sizes across the study (<10), non-parametric alternatives should be used by default.

- Thank you for pointing this out. We have recalculated all statistics using non-parametric tests. Details can be found in the methods section. Please note: since Prism does not provide a to analyze non-parametric data corresponding to a 2-way ANOVA, we transformed the data into ranks and performed a 2-way ANOVA on those ranks based on Scheirer et al, 1976 (this only applied to the body temperature measurements).

2. All statistical results should be clearly reported in the Results section. Given the small sample sizes, it would be better to focus on effect sizes and confidence intervals for interpreting the biological significance of results.

- Thank you for suggesting this. We have calculated the effect sizes for all comparisons (described in methods) and report them either directly in the figures together with the p-values or as a supplementary table (in the case of Figure 4) for clarity. The majority of effect sizes could be considered large (>0.5).

3. According to Table 1, the number of cages per treatment group ranged from 2 to 6, and the number of mice per cage was also highly variable (from 1 to 6). So, for example, looking at Table 1, measures for corticosterone were taken from a singly housed mouse in C1, and averaged from 5 mice in cage C6. When cage means are calculated from different numbers of mice, each cage mean will probably have different variance. I am not sure treating a single mouse is equivalent to the mean of 5 or 6 mice. ANOVA and t-tests assume equal variance across groups, which is probably violated here.

- Please see previous answer regarding statistics above, all statistics have been recalculated with non-parametric tests.

4. Line 298-301 – the authors report that because animals were tested individually after

injections, individual mice were treated as individual units for statistical analysis. Mice from the same home-cage should not be treated as independent even if they were tested individually. Experimental unit should be cage. Mixed models can be used for analysis, where mouse nested in cage is used as a random factor.

- This statement was related to blood draws, not injections. In the injection groups, all mice were tested individually, but with the co-housed animals present in the home cage, and we always used cages as the experimental unit. However, in case of the blood draws, mice were assessed individually without the co-housed animals being and without coming into contact with the co-housed animals between procedure and measurement of the stress parameters. Therefore, individual mice were treated as the experimental unit, equivalent to the general approach with regards to other anxiety tests (eg. open field), where mice are group-housed, but assessed individually without other mice present.

5. I have an additional question about voluntary interaction measurements. The authors measure latency to approach the experimenter's hand. The literature that they cite (Henderson et al 2020, Gouveia et al 2013), that also conducted voluntary interaction tests however, measure differences in interaction time (% time spent interacting with the experimenter's hand during 60s). Can the authors explain why they used a different metric here (latency to start interacting) as the papers they cited?

- Thank you for catching this mistake. We indeed cited the wrong papers for this method. Our approach followed the study of Hohlbaum et al (2020), who measured "latency to first voluntary interaction with the experimenter's hand" to assess "anxiety-related behavior in anticipation of handling". We have replaced the original citations with the correct one.

Also, in the first part of the experiment you measure latency to interact in group housed mice, but in the second experiment you measure it on each individual mouse before it was placed back in the home cage. I don't think either method is better, but the difference in measuring interaction should be more explicitly stated in the discussion as it could have affected the results.

- The latency to interact was also measured inside the home cage after blood sampling, however without the cohoused mice being present. This is also stated in the respective method section. We decided to use this strategy, because the pictures for MGS had to be recorded as immediately as possible after the blood draws, ie. on the Vetbed right after releasing the mice from the tunnel or restrainer. Releasing the mice back into the home cage would have made it challenging to take a picture suitable to assess the MGS. Therefore, we also wanted to record the latency to interact individually instead of in cage groups.

Some additional minor points:
Abstract: the strain and sex of animals tested should be mentioned in the abstract.

- ➢ This has been added

Line 57: It should be clearly stated that the latency to interact with experimenter decreased for both treatment groups, or there was no treatment effect.

- ➢ This has been added.

Line 58: Change "normal behaviour" to "normal activity levels" as you did not measure behaviour but activity (as mentioned in Results, line: 434).

- ➢ Thank you for suggesting this. We have changed this throughout the manuscript

Line 55: The authors mention that body surface temperature was measured, but the result is not mentioned in the abstract. All outcome measures that were assessed should be mentioned in the abstract.

- ➢ This has been added to the abstract

Lines 119-128: this paragraph should go at the beginning of the discussion.

- ➢ We combined this paragraph with the first paragraph of the discussion.

Line 141: It is not clear from the times stated (4pm and 7pm) if experiments were conducted during the light or dark phase.

- ➢ We have added this information

Reviewer #2: Schlutt and colleagues propose a refinement strategy for intraperitoneal injections and blood sampling based on the use of a tunnel in combination with a training protocol. The manuscript addresses an important issue in the context of the 3Rs by proposing a refined strategy for routine procedures in animal facilities. As such, I consider it to be an important addition to the knowledge base on the 3Rs.
Nevertheless, there are several errors and points that require clarification. Specific comments on the different sections are provided below.

Introduction

· On lines 87–88, the authors refer to a "higher risk of painful mistakes due to wrong restraining or application." Please clarify what is meant by these "painful mistakes."

- ➢ We have rephrased this sentence for clarity

· Throughout the manuscript, the term "medical training" is used to describe the training protocol/program. I suggest using a different term, as this wording may be misleading.

- ➢ In veterinary medicine the term medical training is used for training of animals to voluntarily cooperate with medical procedures and examinations with the goal to reduce or eliminate the need for physical restraint and thereby reduce stress. We find

this definition very fitting for the purpose of our training and would therefore like to keep this wording.

• On line 117 and in most of the manuscript, the term “blood draws” is used, whereas the title uses “blood sampling.” I recommend standardizing the terminology throughout the manuscript.

- This has been changed

• The Introduction would benefit from a more thorough review of previously developed refinement methodologies for substance administration in laboratory mice.

- Thank you for this suggestion. We have now added this to the introduction.

Materials & Methods

There is a recurrent lack of detailed information regarding the animals and their housing conditions. In accordance with the ARRIVE guidelines and reproducibility standards, I strongly recommend including the following information:

• Age range of the animals used in the study;
• Details of the cage type (e.g., dimensions, manufacturer);
• Details of the diet (e.g., manufacturer, treatment, product code);
• Details of water provision (e.g., water bottles vs. automated watering system, treatment);
• Details of nesting, bedding, and environmental enrichment (amount, manufacturer, product code).

- We have added the requested information to the methods section

• Line 153 – Please provide more details on the Western-style diet (e.g., percentage of fat, sucrose, protein, moisture content).

- We have added this information in the methods

• Lines 159–160 – The authors state that animal “welfare and health status” were assessed daily. Please describe this procedure. Was this a daily visual health check?

- Yes, visual health checks were performed daily. We have added this in the methods section

• Lines 161–165 – The authors state that “death was not a likely outcome or planned experimental endpoint.” Please clarify whether “death” refers to spontaneous death or euthanasia. Death should be prevented through the application of humane endpoints and should not be considered an endpoint in itself. Moreover, all animals undergoing procedures should be monitored using predefined criteria and humane endpoints at all stages of the experiment.

- Thank you for pointing out that this was not clearly described. Neither spontaneous death nor euthanasia were a likely or planned outcome in this study. We agree, that

death should be prevented through the application of humane endpoints, which was the case in our study. We have clarified these points in the respective section.

• Line 166 – Humane endpoints included weight loss >20%. Why was body condition scoring not included? In oncological models with internal tumors, body condition score is generally more sensitive than body weight for assessing overall condition (see OBSERVE guidelines). Given that this is a refinement-focused manuscript, this omission should be justified.

- We thank the reviewer for pointing this out. In fact, body condition scoring was included in our humane endpoints as we followed the OBSERVE guidelines for our study, but we forgot to mention this in the manuscript. We included weight loss as a separate endpoint independent of the body condition score, because the latter is often difficult to assess in the western-diet fed mice. Due to the obesity, a weight loss of 20% does not necessarily lead to a body condition score of 2 or lower, but is considered a humane endpoint as per local animal welfare regulations. Therefore, both parameters were considered as separate humane endpoints. We have now clarified this in the manuscript.

• Line 167 – Tumor diameter is listed as a humane endpoint. How was tumor size measured?

- Tumor diameters were determined via magnetic resonance imaging

• Table 1 – Table 2 presents an overview of experimental groups, assessed parameters, and the number of animals per cage. This table raises several questions:
o Why were not all parameters assessed in all experimental groups? For example, tracking was assessed only in the CCl_4 groups with medical training and tunnel handling, as well as in the conventional group.

- The purpose of the movement tracking was to prove that our novel injection technique, which consists of both training and use of the tunnel, improves the welfare of the mice after injections. As we have also described in the discussion, we noticed that it is essential for the animals to be introduced to the injection tunnel during the training program in order to facilitate them entering the tunnel without force. Otherwise, trying to convince the animals to enter the tunnel of forcefully placing them inside the tunnel would again result in additional stress for the animals. Therefore, we did not see benefit of assessing either part of the method (training vs. use of the tunnel) by itself.

o Why did the number of animals per cage vary from 1 to 6? Could this variation have influenced parameters such as corticosterone levels?

- Animal numbers differed between cages, because animals entered the experiments at the age of 2 weeks (ie. before weaning), when DEN was injected to induce tumor development. Therefore, the number of animals in each cage was dependent on the number of male pups per litter that could be weaned at the same time and housed in the same cage. Male pups of different ages could not be housed together, because

this would lead to aggressive behavior. In addition, animal facility regulations prevented us from separating male pups of the same cage when no aggressive behavior was apparent, mainly due to space reasons. Hence, we only had very limited influence on the number of animals per cage. It is well known that stress levels differ in single-housed vs. group-housed mice, but all our animals (except 1 untreated control) were group-housed. Therefore, we consider it unlikely that the exact group sizes had an influence on stress parameters, even though we cannot rule this out completely.

• Lines 228–232 – The description of the "conventional method" for intraperitoneal injections does not correspond to the method typically described for scruffing, nor does it match the description in the cited reference. In particular, the method of immobilizing the animal prior to scruffing is unclear. Conventionally, immobilization is achieved by securing the tail and allowing the mouse to grasp the cage grid with its forepaws. Please justify the choice of the described method. Additionally, given that this is a refinement-focused study, the authors should acknowledge that a more refined and widely accepted scruffing method—the three-finger method, which avoids pressure on the neck area—exists and has been described (see Labitt et al., 2021), as illustrated in Figure 2E.

- ➢ Thank you for pointing out that our description of the method was unclear and differed from the cited reference. We do in fact use the method described in the reference and have now added more detail to this section to make this clearer. We are of course aware of the 3-finger method; however, this study was initiated before the study by Labitt et al was published in 2021. As this was a welfare study, we did not want to change the scruffing method in the middle of an ongoing study. The method we use here represents the state of the art in our animal facility at study initiation in 2020. We have added discussion of the 3-finger method to the manuscript.

• Line 266 – A space is missing between "interact" and "as."

- ➢ Thank you, this has been added

• Lines 270–271 – It is stated that "each mouse was assessed individually, but with the co-housed animals present in the home cage." Why was this assessment not performed in complete isolation? Could dominance hierarchies or social interactions have influenced the results?

- ➢ Yes, we agree that it is possible that social interactions in the cage groups may influence the results. We decided to test the animals in the home cage with the other mice present, because this reflects the "real-life" situation that the animals are exposed to during the experiments. Group housing is the standard in our animal facility, not only due to space constraints but also because complete isolation (especially over long periods of time) has been proven to be a chronic stressor for mice. Therefore, after each injection, mice are placed back in the home cage and are immediately exposed to social contact with their cage mates again. We wanted this

to be reflected when assessing whether welfare of our mice could be improved by training and tunnel injections.

• Body temperature measurements (lines 275–280) – Why was the dorsal region selected for temperature assessment? As infrared thermometers measure surface temperature, contact with the handler's hands during scruffing or proximity to the tunnel could have influenced the readings.

- ➢ Temperature was assessed after placing the animals back in their home cages, so they were no longer restrained by scruffing or in the tunnel. Therefore, proximity to the hand or tunnel did not influence the readings. We chose the dorsal region, because this was easy to reach without touching the animals once they were back in the home cage. Measuring the temperature around the eyes or ears, for example, would have required additional restraining of the animals, which we wanted to avoid.

• Subcutaneous tracking chips and movement tracking:
o Why were RFID chips implanted in the caudal abdominal region, left of the linea alba? This placement is not recommended (see Kahnau, 2026) and is close to the intraperitoneal injection site, potentially increasing discomfort compared with dorsal subcutaneous implantation.

- ➢ This decision was based both on internal expertise with the Intellicage System as well as the recommendations by Kahnau et al, who do not actually comment on abdominal placement but caution against dorsal placement too far back, because: "If the transponder is placed too far back (at the back of the spine), […] it may be difficult for the RFID antenna in the IntelliCage corners to read the transponder number." Therefore, Kahnau et al recommend placement in the neck region, however, this was not feasible for us for two reasons. First, the transponders would be in the area where the neck fur had to be scruffed for the conventional injections without the tunnel, and therefore cause additional discomfort. Second, we can confirm from our own experience that the transponders should not be too far away from the receivers. Because our cages contain a rather thick layer of bedding as an enrichment method (to allow the mice to burrow), dorsal placement of the transponders caused them to be too far away from the receivers to be consistently detected. Abdominal placement helped this issue.

o At what age were the transponders implanted?

- ➢ 5 weeks, this information has been added

o Was analgesia provided?

- ➢ No. Animals were anesthetized, but analgesia was not provided. This procedure was approved by the local animal welfare authorities.

• Blood sampling from the tail vein:

o Please provide more details on the “conventional restrainer.”

- We have added the dimensions of the conventional restrainer to the methods section and included a picture of the restrain in Figure S56 A.

o Does the description provided encompass the entire procedure? Were any measures taken to promote vasodilation?

- No, we did not perform any measures to promote vasodilation. The entire procedure is described.

• Pathological examination – Please describe the euthanasia method used. How long after the final intraperitoneal injection was euthanasia performed?

- Mice were sacrificed 72h after the last intraperitoneal injections using isoflurane overdose. We have added this to the methods.

Results

• Line 372 – A space should be inserted between “interact” and “after,” and between “interact” and “was.”

- This has been added

• Lines 372–373 – The authors state that “latency to interact was quite high and very variable in untrained mice,” but these data are not shown in the figure.

- Thank you for pointing this out. We were referring to the first assessment time point in the graph, which displays much larger error bars than the following time points. We have now included data from a group of untrained mice in Suppl. Fig. X, which clearly shows that the LtI remains high without training, even after the mice already know the test. We have added this data as a supplementary figure, rather than in Figure 4, as we felt that this would make the individual graphs too busy and therefore reduce clarity.

• Line 381 – A space should be inserted between “interact” and “after.”

- This has been added

• Figure 4 – This figure would be more informative if it also included data from untrained mice.

- We have added data from untrained mice to suppl fig. X (see also above)

• Line 426 – I suggest replacing “Behavioral assessment” with “Activity assessment,” as no specific behaviors were scored.

- Thank you for this suggestion. We have applied this change throughout the manuscript

• Lines 473–480 (Pathological examination) – Were any additional procedures performed to confirm the absence of tissue damage resulting from the modified technique?

- No, we did not perform additional assessments

• Lines 488–489 – The authors state that "healthy untrained mice" were used to avoid confounding effects of disease induction. Why was this approach not applied to the other experimental groups?

- The main purpose of our study was to improve animal welfare in a disease model with frequent intraperitoneal injections over long periods of time. Therefore, we assessed our animals repeatedly throughout the experimental course, in order to ensure that the benefit of our training program and improved injection method was not lost with increasing numbers of repeated injections. This assessment would not have been possible in untrained mice receiving just a single injection.

• Was the potential effect of the experimenter on the results evaluated or controlled?

- In the training/injection groups, this was not evaluated, because all animals were trained and injected by the same experimenter, who also measured the different stress parameters. Blood draws were performed by 2 investigators together for easier handling, here, we controlled for interobserver variability by both observers scoring the MGS of each mouse separately and averaging those scores.

Discussion

• Lines 549–554 – The discussion of temperature assessment methods is unclear and should be rephrased. Although the authors refer to different methodologies, they used a contactless infrared thermometer (measurements taken at 2–3 mm from the skin). The discussion should also address other non-invasive and accurate temperature assessment methods, such as thermography, as well as invasive alternatives like implanted microchip transponders.

- We have expanded the discussion on different methods of temperature measurement.

• Lines 571–573 – The authors state that only experienced handlers were included in the study but then mention that newly trained researchers reported decreased hesitation. This sentence should be rephrased for clarity.

- We have rephrased this statement

• Line 581 – The authors discuss the influence of observer experience on Mouse Grimace Scale (MGS) scores. Were the observers in this study experienced? How many scorers were involved?

- Yes, both observers were experienced. Every animal was assessed by 2 scorers independently and the scores averaged to reach a final MGS per animal.

• Lines 597–600 – The authors state that a limitation of fecal corticosterone analysis is the need for metabolic cages. This is incorrect, as fecal samples can be collected directly from the home cage.

· Lines 601–602 – Circadian rhythm is mentioned as a limitation of fecal corticosterone analysis; however, this can be mitigated by collecting samples consistently at the same time of day.

- Yes, we are aware that fecal samples can be collected directly form the home cage and that collection at the same time of day may mitigate effects of the circadian rhythm. We did not mean to say that this is impossible to control, but rather that this necessity adds a layer of complication to experiment planning and implementation. In addition, when collecting samples from the home cage, it is not possible to assess when the fecal pellet has been dropped (and therefore how it pertains to the circadian clock) unless the animals are placed in fresh cages directly before the collection periods (which might increase short-term stress levels) or home cages are cleaned from "old" pellets before the collection period (which adds working time for the experimenters and might disturb the animals). Therefore, we had decided to measure corticosterone accumulation in fur hoping to mitigate these challenges (please see also answer below). We have rephrased this part of the discussion.

· Lines 602–607 – Given these known limitations, please justify the choice to quantify corticosterone in fur.

- We had hoped to mitigate some of the challenges described above for corticosterone metabolite measurements in feces by measuring corticosterone levels in fur, assuming that these would be less sensitive to the circadian rhythm but rather accumulate over time. Our hypothesis was that higher levels of corticosterone would be reached in animals experiencing more stress during the course of the experiments. However, this proved not to be true. When planning this study in 2020, we were of course not aware of the study by Colding-Jorgensen et al from 2023, which confirms our observation in a rat model. Otherwise, we might have decided against this method.

We hope that you find our answers satisfactory and look forward to the further evaluation of our manuscript.
