# Supplementary material for: A novel application tunnel in combination with medical training reduces stress induced by frequent intraperitoneal injections and blood draws in mice
Source: PLoS One. 2026 May 7;21(5):e0341404. doi: 10.1371/journal.pone.0341404 (PMC13152133; doi:10.1371/journal.pone.0341404)
Supplement: Attachment — Submitted filename: Schlutt et al_response to reviewers2.pdf [file pone.0341404.s013.pdf]

Thank you for evaluating our manuscript PONE-D-26-00796R1.
Please find our answers to your comments below:

Introduction:
First sentence: I don't think one can back up the claim that rodents are more indispensable than other animal species! They are more commonly used but you are stating that explicitly and with a reference later on in the same paragraph.

- We removed "especially rodents" from the first sentence as we agree that this applies to all laboratory animal species.

Line 121-124 The last sentence summarizes the results and should therefore not be part of the Introduction

- This sentence was removed.

Line 159 "All male mice born in our facility" – is it really true that all male animals born in the entire facility underwent this protocol? Do you have an entire mouse facility dedicated to this line of research?

- We acknowledge that this sentence may be confusing. Our mice are kept in a larger facility shared by many research groups, where we keep our own breedings since we need the pups at 14 days of age (ie. before weaning). All male mice born in the breeding cages of our research groups underwent this protocol. We replaced "facility" with "colony" for clarity.

Tables 1 and 2 take up several pages and they present information in a level of detail that is more suitable for the Supplementary materials section than for the article proper. Please move these tables to the Supplementary materials, and include only a brief summary of them in the Materials and Methods section.

- We have moved the tables to Supplementary Materials. Since the legend of Figure 2 already includes a short description of the training days we did not add another summary of Table 2, but rather extended the figure legend a bit.

Line 270 Latency to approaching a human hand is a relevant parameter to measure in this study, but I don't think that the main issue here is the stress effect on social interaction. Social interaction is not an appropriate description for how mice react to human handling. Animals interact socially with members of their own species, and may also develop a social relationship to individuals of other species (e.g. dogs, cats, horses, farm animals, rats) but that level of relationship is rarely established with a lab mouse. The human hand is the source of stress and discomfort in handling, and the mice are likely to associate the hand with the negative experience of being handled. Mice who have experienced less stressful handling are likely to show a shorter latency to approach the hand (do the mice really interact with the hand?). Please consider this also elsewhere in the manuscript where this concept is addressed.

- We removed the first sentence of this paragraph and ensured that latency to interact is not discussed as a measure of social interaction throughout the manuscript.

Line 333 Please avoid starting a sentence with a digit.

- The sentence was rephrased.

Lines 416-417 What does "No remarkable changes were detectable depending on training success or age" mean? Please reword.

- We have reworded this sentence.

Lines 433-434 "Cage means (average body temperature of all mice in one cage) were treated as the experimental unit.". I think most readers will understand what you mean, but to use precise and correct terminology, the cage is the experimental unit, whereas the average body temperature of all mice in a cage is the datapoint.

- This was rephrased (also in other figure legends where this applied).

Lines 512-518 How can you measure social interaction when the mouse is alone in the cage with no other mice present?

- We measured the latency to interact, this was phrased incorrectly in the figure title and has been adjusted.

Line 566 "variation", not "variety"

- This has been changed.

Line 577 "increasing", not "maximizing"

- This has been changed.

Lines 578-580 Please reword. I suggest "many studies show improved welfare when replacing tail handling by tunnel or cup handling [15, 29] and this beneficial effect remain even when animals are subject to frequent interventions like ip injections.

- We reworded the sentence following your suggestion.

Lines 616-617 I have never seen the word falsify being used in this way. In science, the word falsify stands for either the formal testing of hypothesis, or for researcher manipulation of data. Please reword. Also, you refer to "by various circumstances" but then only give one example.

- We have reworded this part.
